# Editorial Correspondence

**Published:** 1871-11

**Authors:** 


### EDITORIAL CORRESPONDENCE.

### THE VIRGINIA MEDICAL SOCIETY.

We are indebted to one of our most accomplished Associates, of Virginia, for a very interesting letter (of the 18th ult.,) in reference to the late meeting of the Virginia Medical Society. We were pleased also, to be informed by our friend, of the kind interest which was shown THE GEORCIA MEDICAL COMPANION by that body, and hope our journal will continue, by the united efforts of its many friends, to maintain that degree of favor which was manifested towards it on that occasion. We have no doubt, from the letter of our friend, the Society acted wisely and for the best interests of the profession of their State. We will be pleased to notice, more fully, the proceedings of the Society, when furnished the printed transactions. We regret that want of space debars us the pleasure of giving only a few extracts. Speaking of the Society, he says:

"We had a glorious meeting, practical and useful—able essays were read, and a manifest disposition, on the part of the Society, to do all in their power to elevate the standard of medicine. We had a large attendance. The medical profession in Virginia is moving in the right direction, determined to unite, heart and hand, in assailing and denouncing all irregularities of the profession, and to demolish *quackery and charlatanism.* The fact of a medical student, in these days of *cheap* medical education, having a *diploma*, is no evidence that he is either mentally or morally qualified to practice medicine. We intend to put our profession upon the same footing as the merchant, the mechanic, etc. I trust that our effort in this direction will meet with the consideration it deserves, and that the opprobium will no longer rest on our State, that physicians need not be paid, because the law does not care to protect their interests, putting them at the mercy of their debtors

Dr. A. W. Fauntleroy, of Staunton, was elected President for the next year, and Staunton the place for meeting.

Dr. R. S. Payne, of Lynchburg, the retiring President, delivered an able an interesting address, taking bold an strong grounds for maintaining the dignity of the profession. His address was eminently practical and full of common sense.

Dr. Thebault's "Essay on the Malarial Fevers of Eastern Virginia" is worthy of the great minds of the science of medicine not only in this country but in Europe. It will be published with our proceedings, and will be read with great interest.

Dr. Claiborne, of Petersburg, read an interesting essay on dysmenorrhœa, which was well received and highly spoken of.

Dr. Fauntleroy made an interesting report of the epidemics in the valley of Virginia.

The subject of inflammation was discussed the last night of the meeting, and participated in by Drs. Latham, of Lynchburg, Fauntleroy, of Staunton, McDonald, Langhone, Thebault, and others. I regret that I have not the time to write about the respective merits of those participating in the discussion. Suffice it to say, that the discussion was ably conducted and exhibited great research and study on the part of each gentleman.

There was a banquet given at the Norvell House by the Lynchburg Medical Association—which every body hugely enjoyed—when the death of Dr. Maupin, Professor of Chemistry at the University, was announced. When, after suitable resolutions to his memory were adopted, the members retired from the banquet-room. Dr. Maupin was fatally injured by jumping from a carriage while the horses were running away in returning from the Fair Grounds. He was a delegate to the meeting of the State Society. His sad accident greatly marred the pleasure of the meeting. He is a great loss to our State and to the science of Chemistry. * *

